# IgG4-positive plasma cells in nonspecific sialadenitis and sialolithiasis

**DOI:** 10.1038/s41379-022-01089-5

**Published:** 2022-05-23

**Authors:** Elin Peuraharju, Jaana Hagström, Jussi Tarkkanen, Caj Haglund, Timo Atula

**Affiliations:** 1 Department of Oral and Maxillofacial Diseases, University of Helsinki, P.O. Box 41, FI-00014 Helsinki, Finland; 2 Department of Pathology, University of Helsinki, HUSLAB, Helsinki University Hospital, P.O. Box 21, FI-00014 Helsinki, Finland; 3 Department of Oral Pathology and Radiology, University of Turku, Turku, Finland; 4 Research Programs Unit, Translational Cancer Medicine, University of Helsinki, P.O. Box 22, FI-00014 Helsinki, Finland; 5 Department of Surgery, University of Helsinki and Helsinki University Hospital, P.O. Box 20, FI-00014 Helsinki, Finland; 6 Department of Otorhinolaryngology—Head and Neck Surgery, University of Helsinki and Helsinki University Hospital, P.O. Box 263, FI-00029 HUS Helsinki, Finland

**Keywords:** Chronic inflammation, Autoimmune diseases, Pathology

## Abstract

Chronic sclerosing sialadenitis is commonly regarded as a manifestation of IgG4-related disease. We previously found that a high IgG4 expression or IgG4-related disease could accompany nonspecific sialadenitis, whereas chronic sclerosing sialadenitis was not directly associated with IgG4-related disease. Our previous findings lead us to hypothesize that these inflammatory conditions of the submandibular gland signify a continuous progression of disease rather than different disease entities. We, therefore, aimed to determine the presence of IgG4-positivity and genuine IgG4-related disease in a cohort of 165 submandibular gland specimens from patients who underwent surgery due to chronic nonspecific sialadenitis or sialolithiasis. To do so, we re-evaluated histopathological features and divided samples into three groups: (A) nonspecific sialadenitis without known sialolithiasis, (B) sialadenitis with sialolithiasis, and (C) sialolithiasis without sialadenitis. We performed immunohistochemical staining for IgG4, IgG, and CD31, and assessed the Boston consensus statement criteria for IgG4-related disease in IgG4-positive samples. We also reviewed patient records and supplemented follow-up data with a questionnaire among patients with IgG4-positive samples. IgG4-positive plasma cells (range 1–344) were found in 131 samples. Among these, 19 samples were classified as IgG4-positive (≥70 IgG4-positive plasma cells/high-power field). Two IgG4-positive samples were histologically highly suggestive of IgG4-related disease, but only one had a clinically confirmed diagnosis of IgG4-related disease. Our results indicate that patients with sialadenitis and sialolithiasis often present with IgG4-positive lymphoplasmacytic infiltrates, but exceedingly rarely present with genuine IgG4-related disease. In sialolithiasis without sialadenitis, IgG4-positive plasma cells are often absent or appear in small numbers. These results support our hypothesis of a continuum of disease, and indicate that progressive inflammation of the submandibular gland leads to the development of more specific pathological features over time.

## Introduction

Immunoglobulin G4 (IgG4) related disease often manifests at sites in the head and neck frequently affecting the submandibular gland^1^. In the last decade, a specific form of submandibular sialadenitis, known as chronic sclerosing sialadenitis or a Küttner tumor, has often been regarded as a presentation of IgG4-related disease of the submandibular gland^2,3^. However, few previous patient series exist to support this conclusion, specifically among Western countries^1,4^.

Contrary to current consensus, our previous results from a Finnish patient cohort revealed that chronic sclerosing sialadenitis is rarely associated with IgG4-positive plasma cell infiltration. Among 51 patients diagnosed with chronic sclerosing sialadenitis, only two presented with genuine IgG4-related disease. Furthermore, these two specimens did not entirely fulfill the histopathological criteria for chronic sclerosing sialadenitis, but were instead classified as nonspecific sialadenitis^5^. Another significant finding was the presence of elevated numbers of IgG4-positive plasma cells in specimens associated with sialolithiasis and nonspecific forms of sialadenitis. This phenomenon had not been described previously^6,7^.

The finding that IgG4-positive plasma cell infiltrates were not strictly found in chronic sclerosing sialadenitis alone raised the question of whether these inflammatory conditions of the submandibular gland could form part of a disease continuum. Specifically, it remains unclear if nonspecific forms of sialadenitis develop features characteristic of chronic sclerosing sialadenitis or even IgG4-related disease over time. Another area of interest speculates that IgG4 plays a role in this proposed continuum of disease.

In this study, we examined the occurrence of IgG4-positive plasma cell infiltrates and other features characteristic of IgG4-related disease in a comprehensive cohort of submandibular gland samples from patients diagnosed with nonspecific sialadenitis, sialolithiasis, or both.

## Materials and methods

We retrospectively identified patients diagnosed with nonspecific sialadenitis and sialolithiasis from among all patients who underwent submandibular gland surgery between 1 January 2000 and 31 December 2012 in the Helsinki University Hospital catchment area, comprising 1.6 million inhabitants. To do so, we searched the Q-pati database used in the Department of Pathology (HUSLAB) for a diagnosis of sialadenitis or sialolithiasis, and included all patients who underwent submandibulectomy during the study period for histopathologically confirmed nonspecific sialadenitis, sialolithiasis, or both. Patients with diagnoses of Sjögren’s syndrome, sarcoidosis, lymphoepithelial sialadenitis and granulomatous sialadenitis were not included in this study. Tissue samples and hematoxylin and eosin (H&E) stained slides for each sample were acquired from Helsinki Biobank. The local research ethics committee approved the study design, and we received institutional permission to conduct this study (HUS/967/2017).

### Cohort size and patient data

Clinical patient data including sex, age at the time of surgery, and the preoperative diagnosis were collected from the Helsinki University Hospital patient records and pathology reports. Specimens were divided into three groups based on the preoperative diagnosis and the final histopathological diagnosis: (A) nonspecific sialadenitis without known sialolithiasis, (B) sialadenitis with sialolithiasis, and (C) sialolithiasis without sialadenitis. A mild, periductally located inflammatory infiltrate was included in group C. The presence of a sialolith was determined based on either the clinical history or the finding from the histological specimen.

### Histopathological evaluation

Three researchers including two experienced pathologists (EP, JH, and JT), evaluated all histological H&E-stained submandibular gland samples and assessed the histopathological criteria for IgG4-related disease as follows: lymphoplasmacytic infiltrates, presence of lymphoid follicles, storiform fibrosis, obliterative phlebitis, and eosinophilia^8,9^. The degree of fibrosis and density of the inflammatory infiltrates were scored, and presence of acinar atrophy was assessed. All researchers evaluated the samples independently, and if the scores differed between researchers the assessment was made in tandem.

### Immunohistochemistry

The formalin-fixed submandibular gland samples were cut into 4-µm-thick sections and stained for IgG4 using a mouse monoclonal anti-human IgG4 antibody (MCA2098G, BioRad, CA, USA). IgG4-positive specimens with plasma cell infiltrates with ≥70 IgG4-positive plasma cells/high-power field (HPF; x40) were further stained for IgG using a rabbit polyclonal anti-human IgG antibody (A0423, Dako, Glostrup, Denmark) and for CD31 using a mouse monoclonal (Clone JC70A) anti-human CD31 antibody (M0823, Dako, Glostrup, Denmark). Deparaffinization and antigen retrieval were performed in a Pre-Treatment module (Agilent Dako, CA, USA) using a pH 9 retrieval solution (EnVision Flex target retrieval solution, high pH, DM828) for 15 min at 98 °C. The sections were then stained in an Autostainer 480S (LabVision) using the EnVision Flex Detection System (Agilent Dako, CA, USA). Samples were treated with EnVision Flex peroxidase-blocking reagent (SM801) for 5 min. All slides were incubated with mouse monoclonal IgG4 antibody (1:100) for 1 h and the IgG4-positive specimens (≥70 IgG4-positive plasma cells/HPF) were incubated with rabbit polyclonal IgG antibody (1:16,000) and mouse monoclonal CD31 antibody (1:50) for 1 h. Appropriate dilution and incubation time was optimized for each antibody, Dako REAL Antibody diluent was used for antibody dilution. Subsequently all slides underwent 30-min incubation with peroxidase-conjugated EnVision Flex/HRP (SM802) rabbit/mouse (ENV) reagent. Slides were visualized using DAB chromogen (EnVision Flex DAB, DM827) for 10 min. Mayers hematoxylin (S3309, Dako) was used for counterstaining.

### Evaluation of immunohistochemical staining

Based on the IgG4 staining, samples were classified as either IgG4-positive or IgG4-negative by counting the plasma cells immunopositive for IgG4 at three different HPFs (x40) in each specimen. We used the same cut-off point for IgG4-positivity as in our previous study: specimens with ≥70 IgG4-positive plasma cells/HPF were categorized as IgG4-positive^5^ and were included for further study and assessment of genuine IgG4-related disease. To do so, all IgG4-positive specimens were additionally immunostained for IgG, whereupon the IgG4/IgG ratio was calculated after determining the number of IgG-positive plasma cells at three HPFs. For the IgG4-positive samples where obliterative phlebitis could not be excluded based on histological evaluation, CD31 staining was performed. CD31 staining was used instead of elastic stain to assess obliteration of venules as well.

IgG4-postitive specimens were assessed for the probability of genuine IgG4-related disease according to the Boston consensus statement criteria. The Boston consensus statement criteria for the diagnosis of IgG4-related disease in the salivary glands consist of ≥100 IgG4-positive plasma cells/HPF, an IgG4/IgG plasma cell ratio ≥40%, and at least two of three histopathological features. These features are a strong lymphoplasmacytic infiltrate with lymphoid follicle formation; fibrosis arranged, at least focally, in a storiform pattern; and obliterative phlebitis, which, however, is sometimes absent in the salivary glands^8^.

### Follow-up data

Follow-up data for patients with IgG4-positive samples were gathered from patient records and supplemented with a questionnaire. The questionnaire was sent to Finnish-speaking patients with IgG4-positive tissue specimens to determine if any further symptoms related to the salivary glands or other conditions developed following a submandibulectomy. Patient records were reviewed, and the questionnaires were sent to patients in October 2021. The follow-up period was calculated from the time of surgery until the date the questionnaire was returned or the last date when patient records were reviewed. For patients who died, the follow-up period was calculated from the time of surgery until the time of death.

## Results

**Fig. 1:**
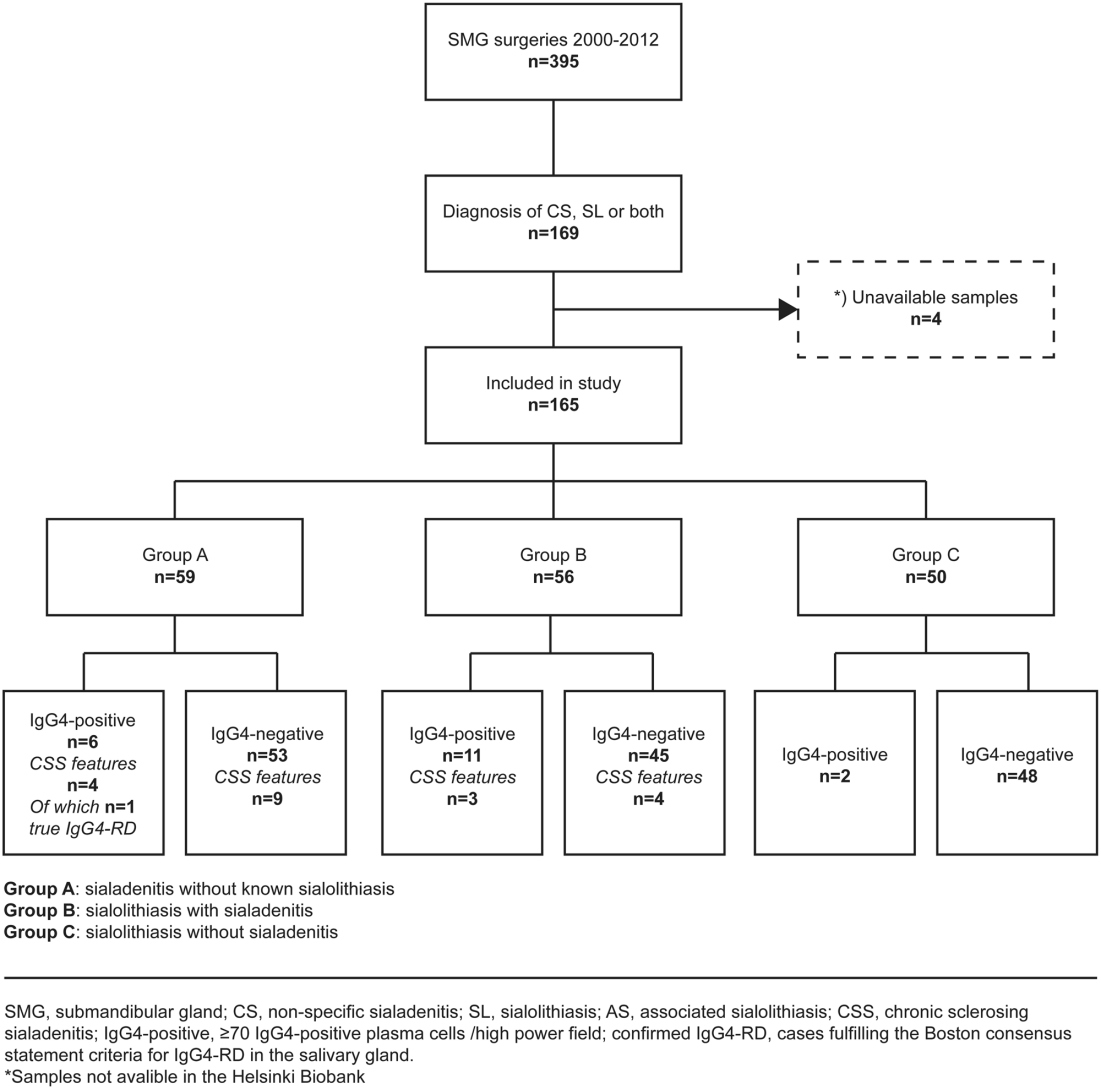
Between 2000 and 2012, a total of 395 submandibular glands were removed from patients within the Helsinki University Hospital catchment area. Among these, 165 patients receiving diagnoses of nonspecific sialadenitis, sialolithiasis, or both were included in this study. These patients were distributed into three groups based on the preoperative diagnosis, the histopathological evaluation, and the immunohistochemical analysis.

During the study period, a total of 395 submandibulectomies were performed in the Helsinki University Hospital catchment area. Among all patients undergoing submandibulectomy during this period, 169 patients (43%) received a histopathological diagnosis of nonspecific sialadenitis, sialolithiasis, or both. From these, we included a total of 165 patients whose samples were available for further study (Fig. 1). None of the included patients overlapped with our previous series which was based on a diagnostic search of chronic sclerosing sialadenitis^5^.

Following histopathological evaluation and reviewing the preoperative diagnoses, 59 patients (36%) were assigned to group A (nonspecific sialadenitis without known sialolithiasis), 56 patients (34%) were assigned to group B (sialadenitis with sialolithiasis), and 50 patients (30%) were assigned to group C (sialolithiasis without sialadenitis). From all patients, 80 (48%) were male and 85 (52%) were female. The median age of patients was 47 years (range 8–86) at the time of surgery.

### Histopathological evaluation and immunohistochemistry

**Fig. 2:**
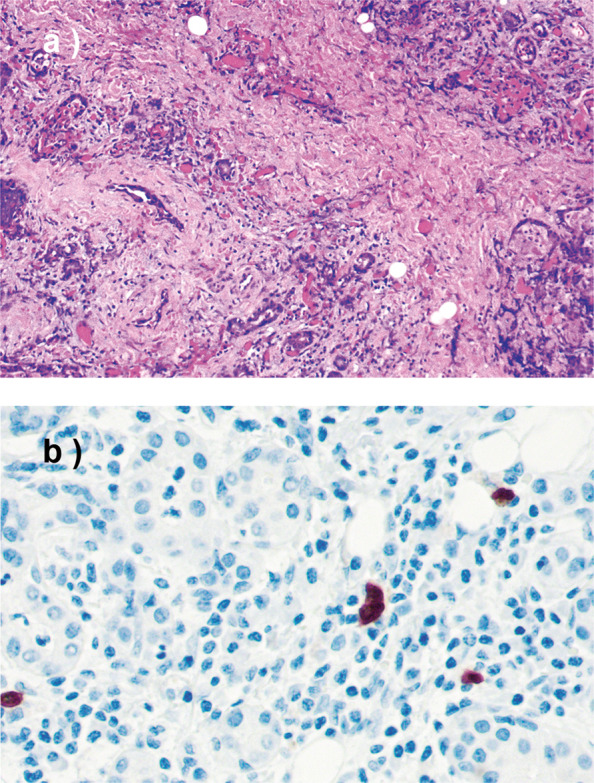
Histological and immunohistochemical features of an IgG4-negative tissue specimen. **a** An IgG4-negative sample meeting stage 4 of Seifert and Donath´s classical criteria for chronic sclerosing sialadenitis. Acinar atrophy and strong fibrosis can be seen in the sample. H&E, ×10. **b** Immunostaining for IgG4 of the same specimen, showing few scattered IgG4-positive plasma cells. ×40.

The evaluation of H&E-stained samples revealed some degree of lymphoplasmacytic infiltrates in all 165 samples, ranging from a localized, mild inflammatory infiltrate to strong lymphoplasmacytic infiltrates affecting the entire gland. In 22 samples (13%), the inflammatory infiltrate could be described as strong. Lymphoid follicles were observed in 57 samples (35%) and acinar atrophy in 47 (28%). Some degree of fibrosis was observed in 107 samples (65%), and in 25 (23%) of these the fibrosis could be described at least focally as storiform. Obliterative phlebitis was not conspicuous upon H&E staining in any of the specimens. Table 1 summarizes the histopathological and immunohistochemical features for each group. After our re-evaluation, 20 samples met either, stage 3 (atrophy of glandular architecture, inflammatory infiltrate with germinal centers, and at least moderate fibrosis) or stage 4 (atrophy of glandular architecture, and strong fibrosis replacing significant parts of the glandular parenchyma) of Seifert and Donath’s classical criteria for chronic sclerosing sialadenitis (Figs. 1 and 2).

**Table 1:** Summary of histopathological and immunohistochemical findings in the three groups: (A) nonspecific sialadenitis without known sialolithiasis, (B) sialolithiasis with sialadenitis, and (C) sialolithiasis without sialadenitis.

	Group A (*n* = 59)		Group B (*n* = 56)		Group C (*n* = 50)	
	*n*	(%)	*n*	(%)	*n*	(%)
H&E staining						
Lymphoplasmacytic infiltrates						
Strong	12	(20)	10	(18)	0	(0)
Moderate	19	(32)	38	(68)	1	(2)
Mild/none	28	(48)	8	(14)	49	(98)
Germinal centers						
Yes	23	(39)	29	(52)	5	(10)
No	36	(61)	27	(48)	45	(90)
Plasma cell infiltration						
Strong	19	(32)	20	(36)	1	(2)
Moderate	17	(29)	33	(59)	14	(28)
Mild/none	23	(39)	3	(5)	35	(70)
Inflammatory infiltrate						
Affects whole gland	21	(36)	28	(50)	5	(10)
Localized	38	(64)	28	(50)	45	(90)
Fibrosis						
Strong	10	(17)	10	(18)	0	(0)
Moderate	23	(39)	42	(75)	22	(44)
Mild/none	26	(44)	4	(7)	28	(56)
Storiform fibrosis	9	(15)	16	(29)	0	(0)
Eosinophilia						
Yes	7	(12)	16	(29)	7	(14)
No	52	(88)	40	(71)	43	(86)
Acinar atrophy						
Yes	21	(36)	25	(45)	1	(2)
No	38	(64)	30	(55)	49	(98)
IgG4 staining						
≥70 IgG4+ plasma cells/HPF	6	(10)	11	(20)	2	(4)
Additional IgG^a^ and CD31^b^ staining						
IgG4/IgG plasma cell ratio ≥ 40%	1	(17)	2	(18)	1	(50)
Obliterative phlebitis	3	(50)	2	(18)	0	(0)

*H&E* hematoxylin and eosin, *HPF* high-power field.

^a^IgG staining was performed only in the 19 specimens showing IgG4-positive plasma cell infiltrates ≥70 IgG4-positive plasma cells/HPF.

^b^CD31 staining was performed only in specimens showing IgG4-positive plasma cell infiltrates ≥70 IgG4-positive plasma cells/HPF and where obliterative phlebitis could not be excluded based on the H&E staining.

**Fig. 3:**
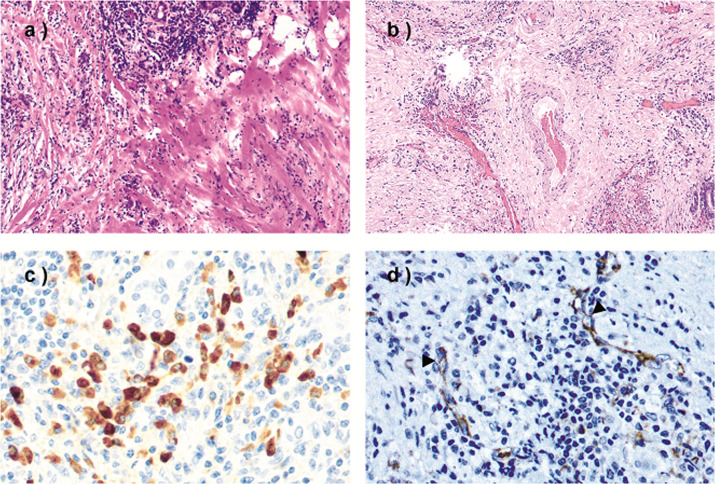
Histological and immunohistochemical features of IgG4-positive tissue specimens. **a** Strong lymphoplasmacytic infiltrates and dense strands of fibrosis visible in the tissue. Degeneration of the acini can also be observed. Hematoxylin and eosin (H&E), ×20, specimen A5. **b** Storiform fibrosis. Lymphocytes and plasma cells are seen interspersed in the fibrotic tissue. H&E, ×10, specimen B1. **c** Immunostaining for IgG4, with abundant IgG4-positive plasma cells. The IgG4 plasma cell count for this field is 90, corresponding to an IgG4/IgG ratio of 0.78 for this hotspot. ×40, specimen A1. **d** Obliterative phlebitis is sometimes absent in IgG4-related disease of the salivary gland. Immunostaining for CD31 shows lymphocytes and plasma cells visible in the lumens of venules (arrowheads). ×40, specimen A1.

We observed IgG4-positive plasma cells in 131 specimens (79%) with a median of 14 IgG4-positive plasma cells/HPF (range 1–344 IgG4-positive plasma cells/HPF). Table 2 summarizes the distribution of IgG4-positive plasma cell infiltrates in the three groups. A total of 19 specimens exhibited ≥70 IgG4-positive plasma cells/ HPF (range 72–344 IgG4-positive plasma cells/HPF) and were classified as IgG4-positive. Four (21%) of the IgG4-positive specimens had an IgG4/IgG plasma cell ratio >40%. Table 3 and Fig. 3 summarize the immunohistochemical, histopathological, and clinical features of the IgG4-positive specimens.

**Table 2:** Summary of the IgG4-positive plasma cell counts and the location of the IgG4-positive plasma cell infiltrates in the three groups: (A) nonspecific sialadenitis without known sialolithiasis, (B) sialolithiasis with sialadenitis, and (C) sialolithiasis without sialadenitis.

	Group A (*n* = 59)		Group B (*n* = 56)		Group C (*n* = 50)	
	*n*	(%)	*n*	(%)	*n*	(%)
IgG4-positive plasma cell count						
0	15	(25)	1	(2)	18	(36)
1–10	21	(36)	16	(29)	20	(40)
11–40	15	(25)	21	(37)	4	(8)
41–69	2	(4)	7	(12)	6	(12)
≥70	6	(10)	11	(20)	2	(4)
Location of IgG4-positive plasma cell infiltrates						
None/sparsely	36	(61)	17	(30)	38	(76)
Throughout gland	21	(36)	23	(41)	2	(4)
Periductal	2	(3)	16	(29)	10	(20)

**Table 3:** Summary of histological, immunohistochemical, and clinical findings for the specimens with ≥70 IgG4-positive plasma cells/HPF in each of the three groups: (A) nonspecific sialadenitis without known sialolithiasis, (B) sialolithiasis with sialadenitis, and (C) sialolithiasis without sialadenitis.

	IgG4-positive plasma cells/HPF	IgG4 plasma cell infiltrates	IgG4/IgG plasma cell ratio	Associated sialolith	Lymphoplasmacytic infiltrates	Germinal centers	Degree of fibrosis	Storiform fibrosis	Eosinophilic infiltrate	Obliterative phlebitis	Age/sex	Probability of IgG4-RD
**Specimen no.**												
** Group A**												
** 1**	**232**	**Throughout gland**	**0.60**	**No**	**Strong**	**Yes**	**Moderate**	**Yes**	**No**	**Yes**	**60/F**	**Confirmed IgG4-RD**
2	154	Throughout gland	0.15	No	Strong	Yes	Moderate	No	No	No	63/F	Insufficient evidence
3	145	Periductal	0.30	No	Strong	Yes	Moderate	No	No	No	47/F	Insufficient evidence
4	101	Throughout gland	0.25	No	Moderate	Yes	Mild	No	No	No	75/M	Insufficient evidence
5	83	Throughout gland	0.09	No	Strong	Yes	Strong	Yes	Yes	Yes (locally)	79/M	Insufficient evidence
6	81	Throughout gland	0.14	No	Strong	Yes	Moderate	Yes	Yes	Yes	57/M	Insufficient evidence
** Group B**												
** 1**	**327**	**Throughout gland**	**0.45**	**Yes**	**Strong**	**Yes**	**Strong**	**Yes**	**Yes**	**Yes**	**67/F**	**Highly suggestive**
2	197	Throughout gland	0.21	Yes	Strong	Yes	Strong	Yes	Yes	Yes	40/M	Insufficient evidence
3	181	Periductal	0.11	Yes	Moderate	Yes	Mild	No	Yes	No	47/M	Insufficient evidence
4	179	Periductal	0.59	Yes	Moderate	Yes	Moderate	No	Yes	No	43/M	Insufficient evidence
5	153	Periductal	0.20	Yes	Moderate	Yes	Moderate	No	Yes	No	54/M	Insufficient evidence
6	114	Periductal	0.14	Yes	Moderate	Yes	Strong	No	No	No	86/F	Insufficient evidence
7	102	Periductal	0.10	Yes	Moderate	No	Moderate	No	No	No	27/F	Insufficient evidence
8	93	Throughout gland	0.14	Yes	Moderate	Yes	Strong	Yes	No	No	37/M	Insufficient evidence
9	91	Throughout gland	0.15	Yes	Moderate	Yes	Moderate	No	No	No	44/M	Insufficient evidence
10	72	Throughout gland	0.06	Yes	Moderate	Yes	Moderate	Yes	Yes	No	32/M	Insufficient evidence
11	74	Periductal	0.10	Yes	Moderate	No	Moderate	No	No	No	48/M	Insufficient evidence
** Group C**												
1	344	Periductal	0.41	Yes	None/mild	No	Moderate	No	Yes	No	56/F	Insufficient evidence
2	92	Periductal	0.23	Yes	None/mild	No	Moderate	No	Yes	No	55/F	Insufficient evidence

Two patients, one with confirmed IgG4-related disease and one with a tissue sample highly suggestive of IgG4-related disease, appear in bold.

*HPF* high-power field.

### Patient follow-up data

The histological and immunological evaluation revealed two patients whose tissue samples were histologically highly indicative of IgG4-related disease (Table 3). In the first patient, the patient record revealed a confirmed IgG4-related disease diagnosis 2 years following a submandibulectomy. Following the diagnosis of IgG4-related disease, the patient was followed at the internal medicine clinic. For the second patient, the patient record revealed no hospital visits related to salivary gland symptoms or symptoms indicative of IgG4-related disease. The second patient, thus, did not receive a clinical diagnosis of IgG4-related disease despite having a tissue sample that fulfilled the Boston consensus statement criteria. In what follows, we describe in detail the clinical data for these two patients with tissue samples highly indicative of IgG4-related disease.

#### Patient one

At the time of the submandibulectomy, the patient presented with swelling of both submandibular and parotid glands, and was preoperatively diagnosed with chronic sialadenitis before undergoing excision of the left submandibular gland. A diagnosis of IgG4-related disease was established 2 years later when the patient presented with pancreatic symptoms. The patient received prednisolone, but persistently showed elevated serum IgG4 levels despite treatment. Eleven years following the IgG4-related disease diagnosis, the patient presented with a suspected IgG4-related pseudotumor of the retroperitoneum. The abdominal mass did not respond to prednisolone treatment and was surgically removed. The lesion was postoperatively identified as a liposarcoma of the retroperitoneum.

#### Patient two

This patient experienced prolonged swelling of both the submandibular and parotid glands, and upon sialendoscopy sialoliths were observed in all of the major salivary glands. The patient received several prescriptions for antibiotics and corticosteroids, but ultimately the right submandibular gland was excised due to persistent symptoms. Following the submandibulectomy, the patient no longer received corticosteroid treatment. Furthermore, the patient records made no mention of IgG4-related disease in any other organ or data on the serum IgG4 levels. Clinically and histologically, the patient received a diagnosis of sialolithiasis. Nine years after the submandibulectomy, a nasal polyp was removed from the patient at our clinic, shortly after which the patient died. During the 9-year period following the submandibulectomy through the patient’s last hospital visit, no follow-up visits regarding the salivary glands were registered.

According to the patient records, none of the 17 remaining patients with IgG4-positive tissue samples showed signs or symptoms indicative of IgG4-related disease.

The questionnaire regarding additional salivary gland symptoms was mailed to 12 of the 19 patients with IgG4-positive specimens. Among the remaining patients, five died during follow-up and two were not Finnish-speaking. In total, 10 of 12 patients (83%) responded to the questionnaire.

The questionnaires revealed symptoms in other salivary glands in three patients. Symptoms included mild intermittent pain and transient swelling during mealtimes, but none of these patients sought treatment for the symptoms reported. The median follow-up time among the 19 patients with IgG4-positivity was 15 years (range 2–19 years).

## Discussion

In our cohort of 165 patients diagnosed with sialadenitis, sialolithiasis, or both, IgG4-positive plasma cells were observed in the majority (*n* = 131) of samples. Quite often (19 of 131), the amount of IgG4-positive plasma cells reached the cut-off point for IgG4-positivity (≥70/HPF). Furthermore, two IgG4-positive samples showed histological and immunological features highly indicative of IgG4-related disease^8^. Of these two patients, one received a diagnosis of IgG4-related disease confirmed following submandibular gland surgery. IgG4-positivity was most frequently found in samples with sialadenitis, both with and without associated sialoliths. In contrast, the absence or only small amounts of IgG4-positive plasma cells were most frequently registered in tissue samples accompanying sialolithiasis without sialadenitis (group C). These results support our previously hypothesized continuum of disease^5^. That is, as opposed to the early stage of disease, more severely affected salivary glands frequently showed more specific pathologic features associated with chronic sclerosing sialadenitis or even IgG4-related disease, such as IgG4-positive plasma cell infiltrates, storiform fibrosis, glandular atrophy, and lymphoid follicles.

IgG4-related disease was recognized at the beginning of the twenty-first century, when pancreatic specimens from patients with type 1 autoimmune pancreatitis were found to contain large amounts of IgG4-positive plasma cells^10^. To date, manifestations of IgG4-related disease have been found in virtually every organ system, and the major salivary glands are among the most affected organs^11–13^. An IgG4-related disease diagnosis is primarily based on the strikingly similar histopathological features observed in the affected organs. These features include strong lymphoplasmacytic infiltrates rich in IgG4-positive plasma cells, obliterative phlebitis, and fibrosis arranged in a storiform pattern^8^. A reliable diagnosis of IgG4-related disease, however, requires a correlation across clinical, radiological, and serological findings in addition to histological findings^14,15^. The precise pathophysiological mechanisms and the etiology underlying IgG4-related disease remain largely unknown.

We divided the samples in our series into three different groups (A–C) based on the presence of inflammatory infiltrates and sialoliths. IgG4-positive plasma cells ranging from 11 to 327/HPF were most frequently identified in specimens from group B, where 70% of the samples showed ≥11 IgG4-positive plasma cells/HPF. In the two other groups (A and C), IgG4-positive plasma cells ranging from 11 to 344/HPF were found in 39% and 24% of the samples, respectively. Previous studies rarely identified IgG4-positive plasma cell infiltrates in nonspecific sialadenitis or sialadenitis with sialolithiasis. Thus, this study is among the first to report the presence of IgG4-positive plasma cells in a large cohort of patients diagnosed with nonspecific sialadenitis or sialolithiasis. Previously, Harrison et al. reported a series of 129 cases of chronic sialadenitis, where >50 IgG4-positive plasma cells/HPF were observed in only three samples. In addition, Harrison et al. identified IgG4-positive plasma cells locally around dilated ducts^7^. In our series, IgG4-positive plasma cell infiltrates were observed equally in the periductal areas and throughout the gland. Previous studies generally distinguished IgG4-related disease of the salivary gland and sialolithiasis as two separate entities^6,16^. This partly agrees with our findings, since one patient with a confirmed IgG4-related disease did not present with an associated sialolith. However, we detected IgG4-positivity in samples from patients with sialoliths as well. Findings somewhat similar to those in our series have, however, also been reported in a small cohort of six patients^4^, and in a case report^17^, where a sialolith was reported in patients with IgG4-related chronic sclerosing sialadenitis. Elevated numbers of IgG4-positive plasma cells have also been reported in other nonspecific chronic inflammatory conditions of the oral cavity, such as apical granulomas, plasma cell gingivitis, and oral lichen ruber planus^18^.

In our cohort, the presence and quantity of IgG4-positive plasma cells correlated with the degree of inflammatory infiltrates. All IgG4-positive samples except those belonging to group C showed either strong or moderate lymphoplasmacytic infiltrates (Table 3). Accordingly, fewer IgG4-positive plasma cells were found in group C, where inflammatory infiltrates were mild or entirely absent in all but one of 50 samples (Table 1). Mild to moderate fibrosis was observed in all groups, although severe fibrosis and fibrosis arranged in a storiform pattern only appeared in groups A and B, the samples of which showed more severe inflammatory lesions than those in group C. In our series, most IgG4-positive samples showed a moderate or strong degree of fibrosis (Table 3), some of which also exhibited histological features typical for chronic sclerosing sialadenitis. These results support our hypothesis that chronic nonspecific sialadenitis, chronic sclerosing sialadenitis, and sialolithiasis are not necessarily different entities, but rather represent a continuum of fibroinflammatory processes affecting the submandibular gland^5,7^.

In our cohort, one of 165 patients had a confirmed IgG4-related disease manifesting in the salivary glands and the pancreas. In another patient with sialolithiasis, the histology revealed findings highly suggestive of IgG4-related disease. But, the patient record revealed no disease manifestation in other organs or any clinical signs of IgG4-related disease during follow-up. IgG4-related disease can thus exist in nonspecific sialadenitis and sialolithiasis, although such a finding is exceedingly rare. In our previous study, we examined the relationship between chronic sclerosing sialadenitis and IgG4-related disease in a cohort of 51 patients, identifying only two patients with genuine IgG4-related disease. Furthermore, roughly one-third of patients originally diagnosed with chronic sclerosing sialadenitis appeared histologically more compatible with a diagnosis of nonsclerosing chronic sialadenitis. This also held for both patients with confirmed IgG4-related disease^5^. The incidence of IgG4-related disease in this patient series agrees with findings from other retrospective studies. Specifically, Harrison et al. found no cases of genuine IgG4-related disease in their cohort of 129 patients with submandibular sialadenitis, while Gunasekara et al. identified three cases of actual IgG4-related disease in their cohort of 137 patients originally diagnosed with chronic sialadenitis^7,19^.

While IgG4-positive plasma cells were identified in 131 samples, a significant number of these samples exhibited only small or negligible amounts of IgG4-positive plasma cells (range 1–10 IgG4-positive plasma cells/HPF) (Table 2). Furthermore, IgG4-positive plasma cells were entirely absent in 34 samples. Samples without IgG4-positive plasma cells, however, also exhibited strong lymphoplasmacytic infiltrates, lymphoid follicles, and varying degrees of fibrosis, and were thus histologically indistinguishable from their IgG4-associated counterparts. This finding suggests different inflammatory antigens trigger separate inflammatory processes underlying sialadenitis, some of which may be associated with the overexpression of IgG4-positive plasma cells and some leading to a more nonspecific inflammatory reaction. These different processes appear independent of sialolith formation, since IgG4-associated inflammatory infiltrates along with non-IgG4-associated strong inflammatory infiltrates were identified both in samples with and without an associated sialolith. The pathophysiological mechanism or antigen resulting in an inflammatory process with the overexpression of IgG4-positive plasma cells remain unclear and requires further research.

The samples for this study were collected over a 12-year period from 2000 through 2012. One strength of our study is that we could gather a large number of samples with sialolithiasis and mild inflammatory lesions, since the standard treatment for sialolithiasis at our institution consisted of submandibulectomy during the early phase of the study period. Over time, the treatment of sialolithiasis shifted toward sialendoscopy with minimally invasive procedures, enabling gland preservation^20–22^. Currently, therefore, it would be difficult to collect a similar patient series. In addition, in our study, we achieved a long follow-up period for IgG4-positive patients, since follow-up from both patient records and questionnaires continued until October 2021. One limitation regarding the follow-up data is that patients who underwent submandibulectomy due to nonspecific sialadenitis and sialolithiasis did not receive regular follow-up care. Thus, our follow-up relies on a questionnaire and a review of patient records. However, we must note that we had access to all hospital records within the entire catchment area. A second limitation lies in the lack of data on serum IgG4 levels in patients whose tissue samples presented high numbers of IgG4-positive plasma cells, since the serum IgG4 measurement was not a routine practice among patients with sialadenitis. Currently, we recommend serum IgG4 measurement for those patients whose tissue samples revealed high levels of IgG4-positive plasma cells.

In conclusion, lymphoplasmacytic infiltrates in the submandibular glands affected by sialadenitis and sialolithiasis can exhibit elevated levels of IgG4-positive plasma cells, although IgG4-related disease in this patient population remains exceedingly rare. The existence of IgG4-positive inflammatory infiltrates appears to represent a part of a continuous inflammatory process in the submandibular gland, most likely a phenomenon distinct from genuine IgG4-related disease.
